# Restoration of Hip Kinematics After Arthroscopy for Femoroacetabular Impingement Syndrome: A 1-Year Evaluation of Gait and Stair Performance

**DOI:** 10.1177/23259671251339777

**Published:** 2025-05-26

**Authors:** Edgar Garcia-Lopez, Ryan T. Halvorson, Aidan J. Foley, Alan L. Zhang, Stephanie E. Wong

**Affiliations:** * Orthopaedic Surgery Department, University of California San Francisco, San Francisco, California, USA; Investigation performed at University of California San Francisco, Orthopaedic Department, San Francisco, California, USA

**Keywords:** femoroacetabular impingement syndrome, kinematics, hip arthroscopy, surgical outcomes, motion analysis

## Abstract

**Background:**

Femoroacetabular impingement syndrome (FAIS) is common in athletes, and the effect of biomechanics and biomechanical alterations after hip arthroscopy continues to be investigated. This study aimed to assess the effect of hip arthroscopy on biomechanical function in patients with FAIS during gait, stair ascent, and stair descent.

**Hypothesis:**

Patients with FAIS would exhibit decreased motion preoperatively compared with contralateral asymptomatic hips, but these differences would be corrected postoperatively and these changes would correlate to improvements in patient-reported outcome measures (PROMs).

**Study Design:**

Case series; Level of evidence, 4.

**Methods:**

Ten patients undergoing hip arthroscopy for FAIS were included and completed PROMs as well as kinematic assessment at baseline and 1 year postoperatively. 3D motion tracking was performed using a 10-camera system while patients performed gait, stair ascent, and stair descent. Joint kinematic parameters were calculated in the sagittal, coronal, and transverse planes for the symptomatic and contralateral side. Peak and valley angles for each joint during each task were compared between limbs using linear mixed-effects models. Significant changes in kinematics were correlated to PROMs.

**Results:**

Preoperatively, the symptomatic hip demonstrated significant deficits in gait and stair ascent compared with the contralateral hip. During gait, hip kinematics increased compared with before surgery with respect to flexion (+7.7°± 7.3°), abduction (+2.4°± 3.2°), and external rotation (ER) (+3.0°± 4.9°) (*P* < .01). During stair ascent, hip abduction (+2.8°± 1.7°) and ER (+2.8°± 5.7°) were significantly increased (*P* < .01). During stair descent, hip flexion (+2.5°± 6.4°), extension (+3.9°± 8.3°), abduction (+2.3°± 2.6°), and ER (+4.8°± 4.3°) were significantly increased (*P* < .01). Significant improvements were seen in patient-reported outcomes, but these did not correlate to hip kinematics.

**Conclusion:**

Hip arthroscopy for FAIS improved hip flexion, abduction, and ER during dynamic tasks such as gait, stair ascent, and stair descent comparable with the contralateral extremity. Additionally, patients reported significant improvement in function and pain at 1 year postoperatively, but these improvements did not correlate with improvements in hip kinematics.

Femoroacetabular impingement syndrome (FAIS) is defined as symptomatic contact between the proximal femur and the acetabulum due to abnormal bony morphology.^9,13^ This abnormal contact is a risk factor for labral and cartilage injury and is thought to contribute to the development of hip osteoarthritis.^1^ Although functional biomechanical parameters are known to be altered in patients with FAIS, the exact role of altered biomechanics in the pathogenesis, diagnosis, treatment, and long-term prognosis for FAIS remains to be elucidated.^7,12^ For example, alterations in hip kinematics and dynamics may lead to altered joint stresses, which may play a role in joint degeneration.^14^

Studies investigating FAIS functional kinematics suggest that patients with FAIS walk with decreased hip extension, internal rotation (IR), and external rotation (ER) compared with asymptomatic peers.^13^ Patients with FAIS are also unable to squat to the same depth as controls.^13^ Although corrective surgical treatment for FAIS is associated with symptomatic improvement, it remains controversial whether this leads to biomechanical improvement and whether biomechanical changes, if present, are actually disease modifying and correlate to patient-reported outcome measures (PROMs).^3,11^

The purpose of this prospective biomechanical study was to evaluate hip, knee, and ankle kinematics during normal gait, stair climb, and stair descent, preoperatively and 1 year postoperatively, in a cohort of patients undergoing arthroscopic hip surgery for FAIS. We hypothesized that the hips with FAIS would exhibit decreased motion preoperatively compared with asymptomatic hips. The secondary hypothesis was that there would be improvement in joint kinematics after hip arthroscopy and these differences would not correlate with improvements in PROMs.

## Methods

### Patients

Patients between the ages of 18 and 55 years undergoing hip arthroscopy for FAIS who agreed to be enrolled in this study were prospectively recruited at a single institution (IRB No. 11-06635). One sports medicine fellowship–trained surgeon with a focus on hip arthroscopy performed all surgical procedures in this study (A.L.Z.). Inclusion criteria consisted of patients diagnosed with FAIS indicated for hip arthroscopy who had cam-type FAI and labral tear with failure of nonoperative treatment, including activity modification and physical therapy. An alpha angle ≥55° was used to indicate cam impingement. Exclusion criteria included hip dysplasia (lateral center-edge angle <25°), osteoarthritis (Tönnis grade >1), hypermobility (Beighton score ≥4), requirement for additional procedures (ie, psoas release, cartilage procedure), and symptomatic contralateral hip with signs of radiographic FAIS.

Preoperative baseline data, surveys, and kinematic data for the operative side and contralateral side were collected before surgery and at the 1-year postoperative time point. Patient demographic characteristics, such as age, sex, and body mass index, were recorded. Patients underwent radiographic evaluation, which included preoperative radiographs of the pelvis in the supine anterior-posterior plane and Dunn lateral 45° views of the affected hip.^4,5^ Patients completed the Hip disability and Osteoarthritis Outcome Score (HOOS), a tool validated for use in patients with FAIS.^10,11,15,18^ The HOOS provides 5 subscale scores: Symptoms, Pain, Activities of Daily Living (ADL), Sport/Recreation, and Qualify of Life (QoL).^16,17^ All data were collected in REDCap (Version 8.1.4).

All surgeries were performed at an ambulatory surgery center. Hip arthroscopy, including labral repair, and femoroplasty was performed using a periportal capsulotomy without closure. Postoperatively, patients were touch-down weightbearing for 2 weeks, progressing to full weightbearing. Rehabilitation included regular physical therapy (hip stability/strength and core strength), impact activities and running beginning at 3 months, and return to full activity and sports at 5 to 6 months after surgery.

### Motion Analysis Data Acquisition

Three-dimensional motion tracking and biomechanical testing were performed using a 10-camera motion-analysis system (VICON; Oxford Metrics) set at 250 Hz. A marker set of 45 retroreflective markers was attached to each patient to create the rigid body segments necessary to capture kinematics. Calibration markers were placed on the head of the first metatarsal, medial and lateral malleoli, medial and lateral femoral epicondyles, and greater trochanter of both left and right lower limbs. Rigid body clusters consisting of 4 markers were placed on the lateral sides of the thigh and shank, and rigid body clusters consisting of 3 markers were placed on the heel shoe counter. Additional tracking markers were attached on left and right acromion, C7 vertebrae, sternal notch, L5/S1 joint, anterior superior iliac spines, iliac crests, and head of the fifth metatarsal. Participants wore nonrestrictive clothing and the same shoe type (running shoe model 880; New Balance) to reduce the effect of shoe type on natural gait.

Marker trajectory and ground-reaction force data were both low-pass filtered with a fourth-order Butterworth filter with cutoff frequencies at 6 Hz and 50 Hz using Visual3D (C-Motion). A musculoskeletal model consisting of 8 segments was created for each patient in Visual3D from their respective standing calibration trial. The pelvis and thorax segments were modeled as cylinders, whereas the thigh, shank, and foot segments were modeled as frusta of cones.

After a 1-second static calibration trial was performed, all calibration markers were removed. Each participant was asked to perform a total of 10 trials of each task with 5 trials per side: fixed speed walking (fixed speed 1.35 m/s), stair ascent, and stair descent.^8^ For the stair activity, the participant was instructed to ascend or descend a 4-step wooden staircase, where each step was 6 inches high. Participants were allowed to practice to become familiar with the protocol. A successful trial was defined as walking within the walking speed window (1.35 ± 0.07 m/s) as well as having the tested limb fall completely within the borders of the force plate especially during stair tasks.

**Figure 1.**
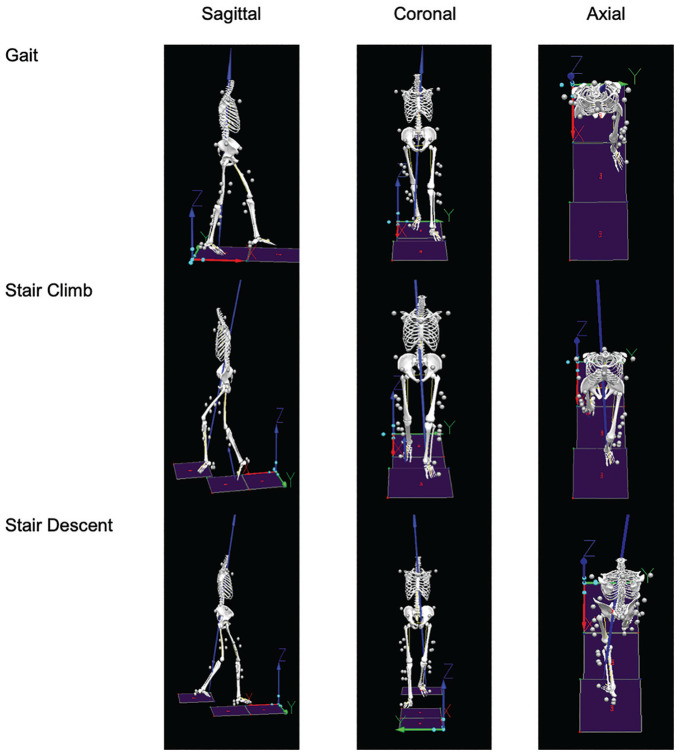
Example of 3-dimensional motion tracking by task.

A local orthogonal coordinate system of the model segments was derived from the standing calibration trial. Segment position and orientation were estimated using an unweighted least-squares optimization. Joint kinematic parameters were calculated using a Cardan rotation sequence in the following order: sagittal (flexion/extension), coronal (abduction/adduction), and transverse (IR/ER) planes. Variables of interest included peak hip, knee, and ankle angles in the sagittal, coronal, and transverse planes at baseline and 1-year postoperative assessments (Figure 1). Peak angles were found by taking the sum of the peaks of 5 independent trials and then averaging them for each individual and repeated for all 3 tasks. For missing values (trials), the mean was taken of the remaining trials for each individual and repeated for all 3 tasks.

### Statistical Analysis

Demographic characteristics were described using means and standard deviations. Peak angles for each joint performing each task were compared between symptomatic and contralateral limbs using linear mixed-effects modeling preoperatively and between baseline and 1-year data for the contralateral side. Values <2° were designated as clinically insignificant, as the value was within the range of measurement error. Postoperative PROMs were compared with preoperative PROMs using paired Student *t* tests. For joint angles and kinematics that significantly improved 1 year after surgery, we then assessed for correlations between joint kinematics and PROM scores. This was done by calculating correlation coefficients between changes in PROM scores pre- to postoperatively with changes in joint kinematics pre- to postoperatively. An a priori power analysis determined that a study population of 9 patients would provide 80% power to detect a 25% change in peak joint hip flexion during gait. A *P* value <.05 was considered statistically significant for all calculations.

## Results

Ten patients (4 women, 6 right-sided affected limbs, mean age 32.1 ± 5.3 years, mean body mass index 24.1 ± 3.3 kg/m^2^) were included who had a mean follow-up of 1.1 ± 0.1 years (Table 1). All PROMs improved at 1 year postoperatively. The largest difference was in the HOOS Sport/Recreation subscale (difference 45.8 ± 12.9; *P* < .001). Significant improvements were seen in the HOOS QoL, ADL, Symptoms, and Pain subscales as well (Table 2).

**Table 1:** Demographic Characteristics of Patients With Femoroacetabular Impingement Syndrome*^*a*^*

Variable	
Age, y	32.1 ± 5.3
Body mass index, kg/m^2^	24.1 ± 3.3
Sex	
Males	60 (6)
Females	40 (4)
Side	
Right	60 (6)
Left	40 (4)

a Data are expressed as mean ± SD or % (n).

**Table 2:** Preoperative and 1-Year Postoperative Scores for HOOS Subscales*^*a*^*

HOOS Subscale	Preoperative	1 Year Postoperative	*P*
Symptoms	56.1 ± 11.5	76.1 ± 10.3	<.001
Pain	56.4 ± 15.1	83.3 ± 9.2	.003
ADL	58.5 ± 17.3	87.7 ± 10.7	<.001
Sport/Recreation	34.7 ± 13.9	80.6 ± 12.1	<.001
QoL	22.9 ± 10.3	64.6 ± 11.7	<.001

a Data are expressed as mean ± SD. ADL, Activities of Daily Living; HOOS, Hip disability and Osteoarthritis Outcome Score; QoL, Quality of Life.

Preoperatively, during gait, significant differences were seen in hip flexion (17.8° vs 21.4°; *P* < .01) (Table 3) between the operative and contralateral hip, respectively. One year postoperatively, the operative hip had significant improvements in hip flexion (+7.7°± 7.3°), abduction (+2.4°± 3.2°), and ER (+3.0°± 4.9°) compared with preoperative findings (*P* < .01) and was in similar state to the contralateral hip. Knee flexion was also lower in the operative side when compared with the contralateral knee preoperatively (40.5° vs 42.9°; *P* = .03), but at 1 year postoperatively the change in knee flexion was not statistically significant. Significant changes in gait joint kinematics from preoperative to 1-year postoperative assessments did not correlate with HOOS subscale scores (*P* > .05).

**Table 3:** Comparison of Joint Kinematics During Gait Between Surgical and Nonsurgical Side Preoperatively and Change at 1 Year Postoperatively*^*a*^*

	Preoperative			1 Year Postoperative	
	Control Side	Operative Side	*P*	Operative Side	*P*
Hip					
Flexion	21.4 ± 8.4	17.8 ± 10.3	<.001*^*b*^*	25.8 ± 5.5	<.001*^*b*^*
Extension	−16.1 ± 8.2	−15.9 ± 9.4	.7	−15.1 ± 6.8	.56
Adduction	7.5 ± 1.7	7.2 ± 2.6	.3	6.2 ± 2.4	.007
Abduction	−2.7 ± 4.0	−1.5 ± 1.8	.003	−3.9 ± 2.8	<.001*^*b*^*
Internal rotation	3.9 ± 5.8	5.7 ± 6.1	.09	4.0 ± 5.8	.05
External rotation	−7.7 ± 5.2	−5.5 ± 5.8	.01*^*b*^*	−8.6 ± 4.3	<.001*^*b*^*
Knee					
Flexion	42.9 ± 8.3	40.5 ± 9.1	.03*^*b*^*	41.8 ± 9.5	.30
Extension	−0.7 ± 3.6	−1.9 ± 5.8	.001	0.7 ± 4.0	<.001*^*b*^*
Ankle					
Flexion	14.0 ± 4.3	13.2 ± 4.0	.03	13.8 ± 3.9	.13
Extension	−12.5 ± 5.2	−11.8 ± 5.8	.8	−12.1 ± 5.0	.78

a Data are expressed in degrees as mean ± SD.

b Difference meets the criteria for clinically significant difference >2.

Preoperatively, during stair ascent, significant differences were seen in hip flexion (39.7° vs 45.6°), IR (4.4° vs 1.7°), and ER (−6.3° vs −8.7°; *P* < .01) (Table 4) between the operative and contralateral hip, respectively. One year postoperatively, the operative hip had significant improvements in hip abduction (+2.8°± 1.7°) and ER (+2.8°± 5.7) compared with preoperative findings (*P* < .01). Preoperative knee flexion was lower on the operative side when compared with the contralateral knee (39.1° vs 46.2°; *P* < .01). At 1 year postoperatively, knee flexion significantly improved (+4.5°± 13.8°; *P* = .02). Significant changes in joint kinematics during stair ascent from preoperative to 1-year postoperative measurements did not correlate to HOOS subscale scores (*P* > .05).

**Table 4:** Comparison of Joint Kinematics During Stair Ascent Between Surgical and Nonsurgical Side Preoperatively and 1 Year Postoperatively*^*a*^*

	Preoperative			1 Year Postoperative	
	Control Side	Operative Side	*P*	Operative Side	*P*
Hip					
Flexion	45.6 ± 8.9	39.7 ± 13.3	>.001*^*b*^*	43.7 ± 10.2	.16
Extension	1.7 ± 7.9	−0.7 ± 9.4	.02	0.2 ± 9.5	.93
Adduction	5.6 ± 3.2	5.7 ± 3.9	.9	5.0 ± 1.9	.12
Abduction	−3.8 ± 2.1	−3.0 ± 2.9	.05	−5.8 ± 1.8	<.001*^*b*^*
Internal rotation	1.7 ± 3.3	4.4 ± 5.6	<.001*^*b*^*	3.7 ± 5.1	.04
External rotation	−8.7 ± 2.6	−6.3 ± 5.3	.002*^*b*^*	−8.2 ± 4.3	<.001*^*b*^*
Knee					
Flexion	46.2 ± 8.9	39.1 ± 14.4	<.001*^*b*^*	43.6 ± 8.9	.02*^*b*^*
Extension	8.4 ± 6.7	3.7 ± 5.9	<.001*^*b*^*	6.1 ± 6.5	.18
Ankle					
Flexion	10.8 ± 5.7	8.8 ± 4.9	.002*^*b*^*	9.1 ± 4.52	.47
Extension	−14.0 ± 7.2	−13.1 ± 6.0	.22	−13.0 ± 5.8	.98

a Data are expressed in degrees as mean ± SD.

b Difference meets the criteria for clinically significant difference >2.

Preoperatively, during stair descent, no significant differences were seen between the operative and contralateral hip (Table 5). One year postoperatively, the operative hip had significant improvements in hip flexion (+2.5°± 6.4°), extension (+3.9°± 8.3°), abduction (+2.3°± 2.6°), and ER (+4.8°± 4.3°) compared with preoperative findings (*P* < .01). Significant changes in joint kinematics during stair ascent from preoperative to 1-year postoperative measurements did not correlate to HOOS subscale scores (*P* > .05).

**Table 5:** Comparison of Joint Kinematics During Stair Descent Between Surgical and Nonsurgical Side Preoperatively and 1 Year Postoperatively*^*a*^*

	Preoperative			1 Year Postoperative	
	Control Side	Operative Side	*P*	Operative Side	*P*
Hip					
Flexion	11.5 ± 10.6	11.5 ± 7.7	.69	14.5 ± 6.7	.009*^*b*^*
Extension	−8.1 ± 12.3	−8.3 ± 12.5	.01	−11.9 ± 10.4	<.001*^*b*^*
Adduction	4.0 ± 3.2	4.5 ± 2.7	.55	3.0 ± 1.8	.002
Abduction	−3.5 ± 3.1	−2.8 ± 2.6	.15	−5.42 ± 1.8	<.001*^*b*^*
Internal rotation	−0.1 ± 2.8	1.6 ± 6.7	.91	−0.8 ± 5.1	<.001
External rotation	−7.2 ± 4.9	−6.1 ± 6.1	.95	−10.8 ± 3.5	<.001*^*b*^*
Knee					
Flexion	48.6 ± 19.3	49.7 ± 20.4	.46	53.5 ± 10.7	.07
Extension	8.6 ± 6.6	9.4 ± 6.1	.77	12.5 ± 8.5	.06
Ankle					
Flexion	27.8 ± 8.6	30.0 ± 5.3	.02^*b*^	29.6 ± 8.5	.62
Extension	−19.4 ± 5.6	−18.7 ± 5.3	.76	−13.8 ± 6.1	<.001*^*b*^*

a Data are expressed in degrees as mean ± SD.

b Difference meets the criteria for clinically significant difference >2.

## Discussion

This study compared kinematics of FAIS patients’ symptomatic limb to their contralateral side at baseline and 1 year after hip arthroscopy. Postoperatively, hip kinematics including flexion, abduction, and ER during gait and stair ascent improved and were comparable to those of the contralateral limb by 1 year postoperatively. Patient's scores on all HOOS subscales also showed significant improvements postoperatively.

During dynamic tasks such as gait and stair ascent, patients with FAIS had limited hip flexion, abduction, and ER when compared with the asymptomatic contralateral limb, which were restored at 1 year after hip arthroscopy. Our findings are consistent with previous studies that examined baseline kinematics during gait and stair ascent in patients with FAIS and compared them with healthy controls at 1 year after surgery.^3,6,19,20^ Rylander et al^19,20^ noted similarly reduced hip flexion at baseline with a significant increase in maximum hip flexion and IR from pre- to postoperative assessments during walking. In our study, we saw reduced ER and abduction at baseline with notable improvement postoperatively, which is consistent with other studies.^6,11^ Contrary to our study, Rylander et al^20^ found that during stair ascent, hip sagittal plane range of motion did not change postoperatively and remained significantly reduced in the FAIS group compared with controls. Maximum hip IR also remained significantly decreased postoperatively.^20^ Stair ascent requires significant hip flexion, which may continue to be hindered postoperatively. It is possible that patients who undergo FAIS surgery do not obtain flexion comparable to healthy controls but still achieve hip flexion, ER, and abduction that are comparable to their contralateral side, as demonstrated in this study. Because ranges of motion can vary between patients, the contralateral, asymptomatic limb may be a better comparison or internal control.

To our knowledge, no other study has examined stair descent in patients with FAIS. We found no difference in these ranges of motion at baseline. Stair descent overall requires less hip and knee flexion than stair ascent, which may account for the lack of difference seen in our cohort. However, at 1 year after surgery, we noted significant improvements in hip flexion, extension, abduction, and ER. Other studies have evaluated and noted significant improvements in postoperative kinematics in tasks such as double-leg squat,^6^ single-leg squat,^21^ and step-down pivot,^2^ which are consistent with our findings as these tasks mirror kinematics for stair descent. Improved range of motion after hip arthroscopy may be a result of decreased mechanical block after femoroplasty and/or improved pain after labral repair.

Knee flexion was noted to be lower on the preoperative side and was restored to that of the contralateral side postoperatively. Limited knee flexion may occur secondary to hip pain or may be a way of compensating while performing dynamic tasks. Most similar studies have focused on hip kinematics.^2,3,6,11,19,20^ However, a recent study examined the kinematics of the pelvis, hip, knee, and ankle joints during performance of a single-leg squat.^21^ Similar to our study, Swindell et al^21^ found that dynamic knee flexion improved after hip arthroscopy, and they also found that this improvement was positively correlated with Hip Outcome Score (HOS)–Sport at both 6 months and 1 year postoperatively. In the current study, we found no significant correlation between knee kinematics and HOOS subscale scores. It is possible that by improving hip kinematics with hip arthroscopy, physiological motion may be restored at other joints.

Patient-reported outcomes as measured by HOOS demonstrated significant improvements 1 year after hip arthroscopy surgery, but did not correlate with joint kinematics. Our analysis revealed that the changes in PROMs after hip arthroscopy were much greater than the changes in kinematics.^11^ Therefore, although positive changes in both PROMs and kinematics were found, the lack of correlation in these analyses was due to the difference in degree of improvement. The HOOS values achieved in this study were well above previous calculated minimal clinically important differences (MCIDs) for a similar patient cohort.^11^ Previous studies have evaluated other PROMs including the HOS and found a significant improvement after hip arthroscopy for FAIS.^11,12,21^ Compared with the current findings, Swindell et al^21^ found a correlation between sagittal hip range of motion during single-leg squat and HOS-ADL at 6 months postoperatively, but Kannan et al^11^ did not find a correlation between HOOS and hip kinematics during gait. It is possible that hip kinematics may not correlate to all PROMs.

The current study is not without limitations. First, the study size was limited to 10 patients operated on by a single surgeon at a large academic institution, which may not generalize to all surgeons’ experience and patient population outcomes. A priori power analysis determined that 9 hips would provide 80% power to detect a 25% change in peak joint flexion. A larger, more diverse patient cohort with multiple surgeons would provide more generalizable data. Second, observed changes in kinematics were small. It is possible that these changes are not clinically significant. Of note, no previous studies have described MCID for kinematic changes after hip arthroscopy. Third, this study measured the HOOS and its subscales, which improved significantly and met MCID at 1 year postoperatively but did not correlate to joint kinematics. It is possible that no correlation was observed due the small sample size, small changes in kinematics, and/or gait kinematics, which may better correlate with other PROMs. Fourth, although we included biomechanics for the asymptomatic, contralateral limb that served as the patient's internal control, it is possible that having a symptomatic hip may affect the biomechanics of the asymptomatic, contralateral hip. Fifth, we focused on 3 tasks (gait, stair ascent, and stair descent) that we deemed clinically appropriate and highlighted only unidirectional movement (sagittal). Further studies should focus on complex coupled movements that may better assess coronal (adduction/abduction) and transverse (IR/ER) plane motion.

## Conclusion

Hip arthroscopy for FAIS improved hip flexion, abduction, and ER during dynamic tasks such as gait, stair ascent, and stair descent at 1 year after surgery. Patients reported significant improvement in pain, function, and quality of life, but these improvements did not correlate to improvements in hip kinematics.

## References

[1] Agricola R Kemp J Waarsing J , et al. Femoroacetabular impingement syndrome is associated with development of hip osteoarthritis within 10-years follow-up: data from the check cohort. Osteoarthritis and Cartilage. 2019;27:S57. doi:10.1016/j.joca.2019.02.081

[2] Alter TD Wichman DM Fenn TW , et al. Hip and pelvis movement patterns in patients with femoroacetabular impingement syndrome differ from controls and change after hip arthroscopy during a step-down pivot-turn task. Orthop J Sports Med. 2024;12(2):23259671231169200. doi:10.1177/23259671231169200 PMC10867405 38361996

[3] Brisson N Lamontagne M Kennedy MJ Beaulé PE. The effects of cam femoroacetabular impingement corrective surgery on lower-extremity gait biomechanics. Gait Posture. 2013;37(2):258-263. doi:10.1016/j.gaitpost.2012.07.016 22939410

[4] Byrd JWT . Femoroacetabular impingement in athletes: current concepts. Am J Sports Med. 2014;42(3):737-751. doi:10.1177/0363546513499136 23982400

[5] Clohisy JC Beaulé PE O’Malley A Safran MR Schoenecker P. AOA symposium. Hip disease in the young adult: current concepts of etiology and surgical treatment. J Bone Joint Surg Am. 2008;90(10):2267-2281. doi:10.2106/JBJS.G.01267 18829926

[6] Cvetanovich GL Farkas GJ Beck EC , et al. Squat and gait biomechanics 6 months following hip arthroscopy for femoroacetabular impingement syndrome. J Hip Preserv Surg. 2020;7(1):27-37. doi:10.1093/jhps/hnaa004 32382426 PMC7195932

[7] Diamond LE Wrigley TV Bennell KL Hinman RS O’Donnell J Hodges PW. Hip joint biomechanics during gait in people with and without symptomatic femoroacetabular impingement. Gait Posture. 2016;43:198-203. doi:10.1016/j.gaitpost.2015.09.023 26475761

[8] Gait Analysis: Normal and pathological function. J Sports Sci Med. 2010;9(2):353.

[9] Griffin DR Dickenson EJ O’Donnell J , et al. The Warwick agreement on femoroacetabular impingement syndrome (FAI syndrome): an international consensus statement. Br J Sports Med. 2016;50(19):1169-1176. doi:10.1136/bjsports-2016-096743 27629403

[10] Hartwell MJ Soriano KKJ Nguyen TQ Monroe EJ Wong SE Zhang AL. Patient-reported outcome surveys for femoroacetabular impingement syndrome demonstrate strong correlations, high minimum clinically important difference agreement and large ceiling effects. Arthroscopy. 2022;38(10):2829-2836. doi:10.1016/j.arthro.2022.03.023 35367302

[11] Kannan AS Hartwell MJ Grace T , et al. Correlating biomechanical gait analysis with patient-reported outcomes after hip arthroscopy for femoroacetabular impingement syndrome. Orthop J Sports Med. 2022;10(9):23259671221121352. doi:10.1177/23259671221121352 PMC9449515 36089924

[12] Kennedy MJ Lamontagne M Beaulé PE. Femoroacetabular impingement alters hip and pelvic biomechanics during gait: walking biomechanics of FAI. Gait Posture. 2009;30(1):41-44. doi:10.1016/j.gaitpost.2009.02.008 19307121

[13] King MG Lawrenson PR Semciw AI Middleton KJ Crossley KM. Lower limb biomechanics in femoroacetabular impingement syndrome: a systematic review and meta-analysis. Br J Sports Med. 2018;52(9):566-580. doi:10.1136/bjsports-2017-097839 29439949

[14] Ng KCG Lamontagne M Labrosse MR Beaulé PE . Hip joint stresses due to cam-type femoroacetabular impingement: a systematic review of finite element simulations. PLoS One. 2016;11(1):e0147813. doi:10.1371/journal.pone.0147813 PMC4727804 26812602

[15] Nguyen TQ Friedman JM Flores SE Zhang AL. Fast starters and slow starters after hip arthroscopy for femoroacetabular impingement: correlation of early postoperative pain and 2-year outcomes. Am J Sports Med. 2020;48(12):2903-2909. doi:10.1177/0363546520952406 32931329

[16] Nilsdotter A Bremander A. Measures of hip function and symptoms: Harris Hip Score (HHS), Hip Disability and Osteoarthritis Outcome Score (HOOS), Oxford Hip Score (OHS), Lequesne Index of Severity for Osteoarthritis of the Hip (LISOH), and American Academy of Orthopedic Surgeons (AAOS) Hip and Knee Questionnaire. Arthritis Care Res (Hoboken). 2011;63(suppl 11):S200-207. doi:10.1002/acr.20549 22588745

[17] Nilsdotter AK Lohmander LS Klässbo M Roos EM. Hip Disability and Osteoarthritis Outcome Score (HOOS)—validity and responsiveness in total hip replacement. BMC Musculoskelet Disord. 2003;4:10. doi:10.1186/1471-2474-4-10 12777182 PMC161815

[18] Oji NM Jansson H Bradley KE Feeley BT Zhang AL. Comparing patient-reported outcome measurements for femoroacetabular impingement syndrome. Am J Sports Med. 2021;49(6):1578-1588. doi:10.1177/0363546521999403 33739894

[19] Rylander JH Shu B Andriacchi TP Safran MR. Preoperative and postoperative sagittal plane hip kinematics in patients with femoroacetabular impingement during level walking. Am J Sports Med. 2011;39(suppl):36S-42S. doi:10.1177/0363546511413993 21709030

[20] Rylander J Shu B Favre J Safran M Andriacchi T. Functional testing provides unique insights into the pathomechanics of femoroacetabular impingement and an objective basis for evaluating treatment outcome. J Orthop Res. 2013;31(9):1461-1468. doi:10.1002/jor.22375 23625839

[21] Swindell H Wichman DM Guidetti M Chahla J Nho SJ Malloy P. Association of changes in hip and knee kinematics during a single-leg squat with changes in patient-reported outcomes at 6 months and 1 year after hip arthroscopy. Am J Sports Med. 2023;51(13):3439-3446. doi:10.1177/03635465231202025 37822105

